# Factors influencing the efficacy of recombinant tissue plasminogen activator: Implications for ischemic stroke treatment

**DOI:** 10.1371/journal.pone.0302269

**Published:** 2024-06-06

**Authors:** Jan Víteček, Andrea Vítečková Wünschová, Sandra Thalerová, Sumeet Gulati, Lukáš Kubala, Michaela Capandová, Aleš Hampl, Robert Mikulík

**Affiliations:** 1 International Clinical Research Center, St. Anne’s University Hospital Brno, Brno, Czech Republic; 2 Biophysics of Immune System, Institute of Biophysics of the Czech Academy of Sciences, Brno, Czech Republic; 3 Department of Anatomy, Faculty of Medicine, Masaryk University, Brno, Czech Republic; 4 Department of Biochemistry, Faculty of Science, Masaryk University, Brno, Czech Republic; 5 Department of Histology and Embryology, Faculty of Medicine, Masaryk University, Brno, Czech Republic; American University, ARUBA

## Abstract

Intravenous thrombolysis with a recombinant tissue plasminogen activator (rt-PA) is the first-line treatment of acute ischemic stroke. However, successful recanalization is relatively low and the underlying processes are not completely understood. The goal was to provide insights into clinically important factors potentially limiting rt-PA efficacy such as clot size, rt-PA concentration, clot age and also rt-PA in combination with heparin anticoagulant. We established a static *in vitro* thrombolytic model based on red blood cell (RBC) dominant clots prepared using spontaneous clotting from the blood of healthy donors. Thrombolysis was determined by clot mass loss and by RBC release. The rt-PA became increasingly less efficient for clots larger than 50 μl at a clinically relevant concentration of 1.3 mg/l. A tenfold decrease or increase in concentration induced only a 2-fold decrease or increase in clot degradation. Clot age did not affect rt-PA-induced thrombolysis but 2-hours-old clots were degraded more readily due to higher activity of spontaneous thrombolysis, as compared to 5-hours-old clots. Finally, heparin (50 and 100 IU/ml) did not influence the rt-PA-induced thrombolysis. Our study provided *in vitro* evidence for a clot size threshold: clots larger than 50 μl are hard to degrade by rt-PA. Increasing rt-PA concentration provided limited thrombolytic efficacy improvement, whereas heparin addition had no effect. However, the higher susceptibility of younger clots to thrombolysis may prompt a shortened time from the onset of stroke to rt-PA treatment.

## Introduction

Stroke, the second most significant cause of mortality and morbidity worldwide, is often associated with long-term disability and notably a huge socioeconomic burden [1]. Approximately 87% of stroke cases are ischemic, resulting mostly from vessel occlusion due to thromboembolic clots [2].

Treatment priority in acute ischemic stroke involves reopening the occluded artery (recanalization). Currently, intravenous thrombolysis by recombinant tissue plasminogen activator (rt-PA) and/or endovascular therapy (mechanical thrombectomy) both present clinically approved treatment methods [3]. Although mechanical thrombectomy is superior to intravenous thrombolysis due to the higher recanalization rate, intravenous thrombolysis remains the first-line treatment, largely due to comparatively low cost and the simplicity of administration. The intravenous thrombolysis by rt-PA has been proven to be an effective option within the 4.5-hour therapeutic window following the onset of stroke in the unselected patient population [4, 5].

The action of rt-PA mediates the conversion of clot-bound plasminogen into plasmin, which is then able to cleave the fibrin mesh of the clot, ultimately resulting in clot degradation followed by the release of fibrin degradation products together with clot-associated blood elements [6]. Successful recanalization of large vessels due to rt-PA-induced thrombolysis remains relatively low (30–40%) [7–10].

Detailed mechanisms behind inter-individual differences in rt-PA efficacy are not completely understood and limit the further improvement of intravenous thrombolysis. Several factors, that affect the efficacy of rt-PA have been identified in clinical practice such as clot size, the timing of administrations and others that affect safety such as the dose of rt-PA or possible co-administration of heparin: Two clinical studies showed that occlusion longer than 8 mm was hard to be cleared by rt-PA [7, 8]. Furthermore, rt-PA dose variation did not change the clinical outcome [11] which was later confirmed by a randomized clinical study comparing standard and lower rt-PA doses [12]. Clinical data concluded that early vessel reopening, which is critically important for good clinical outcome, has been negatively correlated with the time between stroke onset to rt-PA administration [13–15]. Hence, the role of clot age could be significant. Finally, heparin has been used for a long time to treat ischemic stroke but this practice is being questioned extensively [16, 17]. However, mechanisms and the extent of their impact on rt-PA-induced clot lysis have not yet been fully elucidated.

Therefore, in this *in vitro* study, we investigated rt-PA efficacy as a function of clot size, rt-PA concentration, combination of rt-PA with heparin, and clot age. The study has brought novel insights into mechanisms of rt-PA limitations using a single model based on clots prepared out of human blood. Further, it provided implications for observed clinical phenomena concerning treatment of the ischemic stroke.

## Materials and methods

This is an *in vitro* study using an established static thrombolytic model to identify how clot size, rt-PA concentration, clot age and combination of rt-PA with heparin influence thrombolysis. The model consisted of a clot incubated in a plastic test tube and clot degradation was determined by two methods–clot mass loss and RBC release [18].

The study was approved by Ethics Committee of the St. Anne’s University Hospital in Brno (reference number 15V/2017). All healthy volunteers who donated blood into this study provided written informed consent. The recruitment of volunteers started after 7^th^ of March 2017 and finished by 30^th^ of June 2019.

### rt-PA, heparin and incubation media

The rt-PA (Actilyse, provided by Boehringer-Ingelheim International GmbH, Germany; Z. Nr. 1–24,717, 580 000 IU/mg) was dissolved in distilled water to a concentration of 1 mg/ml and was stored aliquoted at -20°C (not re-frozen once thawed). The final concentration of rt-PA was in line with the clinically relevant dosing for patients with ischemic stroke, according to the manufacturer’s instructions and supporting pharmacokinetic data: The recommended initial bolus (10% of the dose) together with one-hour-long infusion of the remaining rt-PA can maintain a steady level of 1.3 mg/l in an average patient (80 kg, 5.5 l of blood) [19]. Heparin (Heparin 5000 IU/ml, Zentiva, Czech Republic) was used at the final concentration of 50 and 100 IU/ml. Such a level is about 100-fold higher than the indicated for the prevention of thromboembolic diseases in patients [20].

All media were prepared using reagent-grade chemicals. Physiological buffered saline (PBS, pH 7.4) contained 8 g NaCl, 2.3 g Na_2_HPO_4_.12H_2_O, 0.2 KCl and 0.2 g KH_2_PO_4_ per litre. Citrated blood was achieved with the addition of 3.8% sodium citrate in the standard ratio (9:1), heparinized blood with heparin in the concentration of 50 IU/ml (Heparin 5000 IU/ml, Zentiva, Czech Republic). Plasma was prepared from citrated blood by centrifugation (1000 g, 10 min).

### Clots preparation and incubation

Human blood was donated by healthy adult volunteers (n = 16), who provided informed consent and had not taken any medication (aspirin, antibiotics etc.) for at least two weeks prior to blood drawing. Clots that were prepared from those blood donors who did not respond to rt-PA treatment (about 30%), were excluded. Haemolytic blood was excluded. Each experiment was repeated three times, each time with an independent donor. All *in vitro* assays were carried out in triplicates.

Clots were prepared from 100, 200, 300 or 400 μl of whole blood without the addition of anticoagulants, for 2 and 5 hours, at room temperature. This resulted in a clot size of 30, 50, 90, and 150 μl, respectively (see S1 Fig). Pyrex glass tubes (internal diameter 8 mm) were used. This glass was already shown to allow consistent clot retraction [21, 22]. The size and shape of clots were carefully checked. Atypical clots were excluded. Two-hours-old clots showed mild retraction whereas 5-hours-old clots were considered highly retracted (S2 Fig).

Each clot was gently washed with 2 ml of PBS buffer to clear the clot of residual serum. After the removal of excess liquid using a cotton wool pad for 10 s, individual clot weights were determined and recorded. Each clot was separately incubated in fluid totalling 500 μl, in a 1.5 ml plastic tube (Eppendorf, Germany), which was placed into a dry-block incubator at 37°C. Incubation lasted an hour–the same amount of time indicated for rt-PA (Actilyse, Boehringer Ingelheim, Germany) treatment of patients with ischemic stroke. Immediately after incubation, clots were blotted of liquid excess and weight was determined as described above. Clots were incubated either in plain medium (control, to determine spontaneous thrombolysis) or with rt-PA (to determine rt-PA-induced thrombolysis).

### Thrombolysis measurement

#### Determination of mass loss

Mass loss was determined by measuring initial and after-treatment clot weight [18]; and was expressed as a percentage of the initial weight. To express net rt-PA-induced mass loss, the control value (spontaneous thrombolysis) was subtracted (mass loss against control).

#### Determination of RBC release

The release of RBCs was based on the spectrophotometric determination of haemoglobin [18]. 5 μl of the incubation medium, immediately after clot incubation were diluted 40 times with PBS. Absorbance was read at 575 nm and the turbidity (absorbance at 700 nm) was subtracted. RBC release was expressed as haemoglobin absorbance (A_575_); to express net rt-PA-induced RBC release, the control value (spontaneous thrombolysis) was subtracted (A_575_ against control). Haemolysis, which can interfere with the determination of RBC release, was assayed after centrifugation of the incubation medium at 4°C 150 g for 10 min, as above. If present, it was under 3% of the total absorbance of released RBCs.

### Data processing and statistics

Data were processed in Microsoft Excel (2016) and are expressed as mean ± standard deviation (SD). The statistical analysis was performed in STATISTICA 12 (StatSoft, Dell Inc., USA). The box plots show the mean value (square), the median (line), the interquartile range (box), and the minimum and maximum values (whiskers). Unpaired t-test and one-way ANOVA with post-hoc Fischer’s LSD test were used to compare the data. P-values ≤ 0.05 were considered to be statistically significant.

## Results

### Stability of clot in different media

Three types of *in vitro* incubation conditions were selected to determine spontaneous thrombolysis: PBS, 0.9% NaCl and heparinized human blood. Mass of the 5-hours-old 50 μl clots spontaneously decreased within the one-hour incubation period as follows: 0.9% NaCl 15.2±4.5%, PBS 18.2±7.0%, and heparinized blood 17.0±6.4% (S1 Table). There were no statistically significant differences among the conditions mentioned (PBS vs. 0.9% NaCl p = 0.23, PBS vs. heparinized blood p = 0.62, 0.9% NaCl vs. heparinized blood p = 0.47). Given the advantages of the experimental setup (simple incubation medium, stable pH, and the possibility to measure released RBCs; as opposed to incubation in heparinized blood), subsequent experiments were carried out in PBS (S1 File).

### Clot size

**Fig 1:**
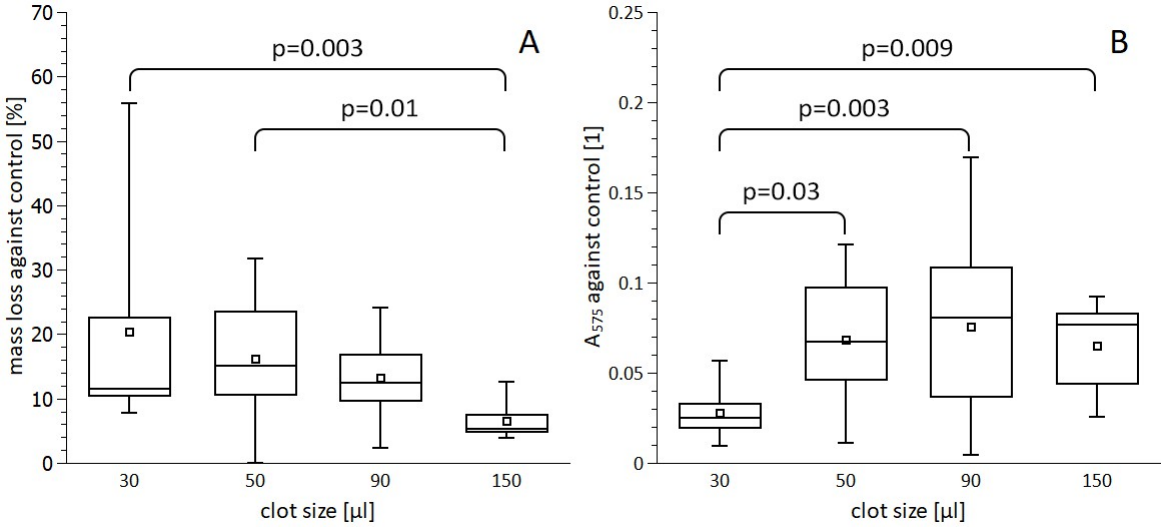
rt-PA-induced lysis of different clot sizes. Clots of indicated size were aged for 5 hours and treated with 1.3 mg/l rt-PA in PBS for 1 hour. Clot degradation was expressed as a mass loss against control (A) and release of red blood cells–haemoglobin absorbance (B). The box plots (square–mean, horizontal line–median, box–interquartile range, whisker–data range) represent data out of at least three biological replicates each containing three parallels. Significant differences (ANOVA, p < 0.05) are indicated. Further p values: 30 vs 50 μl p = 0.28, 30 vs 90 μl p = 0.07, 50 vs 90 μl p = 0.34 and 90 vs 150 μl 0.09 in (A) and 50 vs 90 μl p = 0.58, 50 vs 150 μl p = 0.82, 90 vs 150 μl 0.49 in (B).

The rt-PA induced relative clot mass loss against control was 20.4±16.1% for 30 μl clots as compared to 16.2±9.5%, 13.2±6.0%, 6.5±2.7% of 50, 90, and 150 μl clots (p = 0.28, p = 0.07, p = 0.003, respectively). A gradual decrease of rt-PA-induced thrombolysis with increasing clot size occurred (Fig 1A, S2 Table). Further determination of RBC release showed that 30 μl clots lost significantly fewer RBCs (0.03±0.01) than 50, 90, and 150 μl clots (0.07±0.03, 0.08±0.05, 0.07±0.03; p = 0.009, p = 0.003, p = 0.03; respectively). There were, however, no remarkable differences in RBC release among 50, 90, and 150 μl clots (50 vs 90 μl p = 0.58, 50 vs 150 μl p = 0.82, 90 vs 150 μl p = 0.49; Fig 1B, S2 Table).

### rt-PA concentration

**Fig 2:**
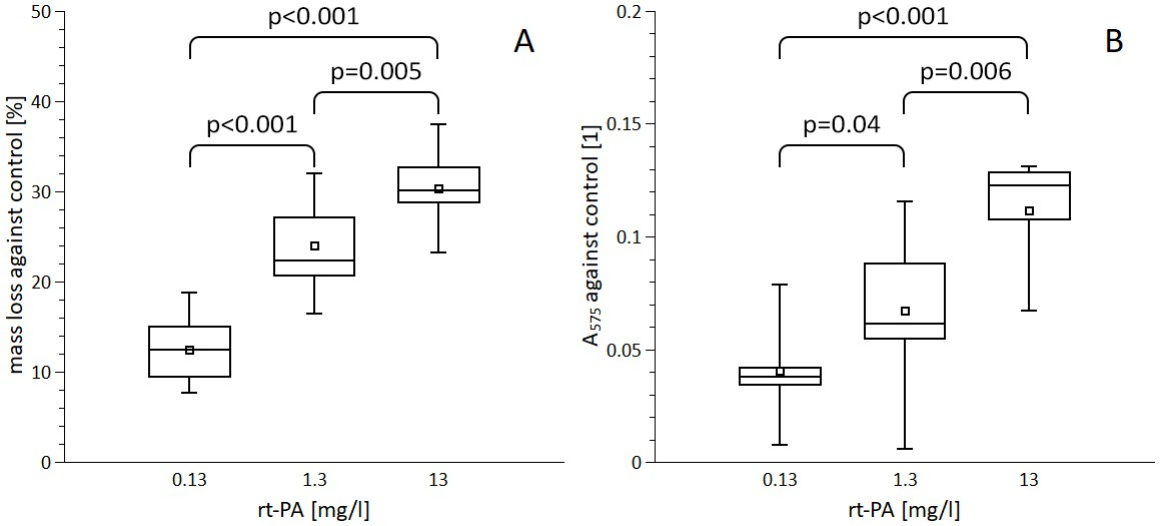
The dependency of thrombolysis on rt-PA concentration. Clots with size 50 μl were aged for 5 hours and treated with indicated concentration of rt-PA in PBS for 1 hour. Clot degradation was determined by mass loss (A) and by the release of red blood cells–haemoglobin absorbance (B). The box plots (square–mean, horizontal line–median, box–interquartile range, whisker–data range) represent data out of at least three biological replicates each containing three parallels. Significant differences (ANOVA, p < 0.05) are indicated.

Concentrations 0.13, 1.3 and 13 mg/l showed a dose-dependent effect on the clot mass loss against control: 0.13 mg/l 12.5±4.1%, 1.3 mg/l 24.0±4.8%, 13 mg/l 30.3±4.0% (0.13 vs 1.3 mg/l p<0.001, 0.13 vs 13 mg/l p<0.001, 1.3 vs 13 mg/l p = 0.005; Fig 2A, S3 Table). A tenfold decrease or increase in rt-PA concentration induced only a 2-fold decrease or increase in clot degradation. Determination of RBC release showed essentially the same course: 0.13 mg/l 0.04±0.02, 1.3 mg/l 0.07±0.03, 13 mg/l 0.11±0.03 (0.13 vs 1.3 mg/l p = 0.04, 0.13 vs 13 mg/l p<0.001, 1.3 vs 13 mg/l p = 0.006; Fig 2B, S3 Table).

### Clot age

**Fig 3:**
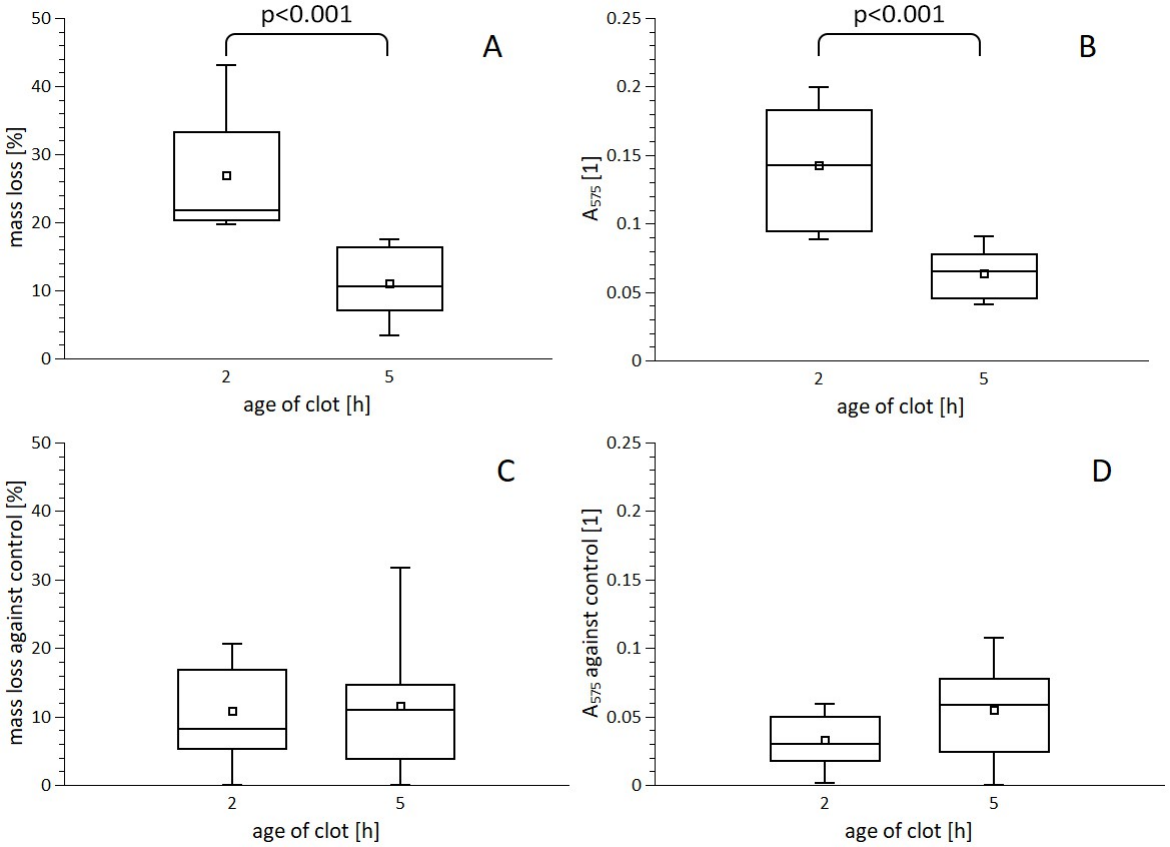
Degradation of clots of different ages. Clots with size 50 μl were aged for indicated times and incubated in PBS (A, B) or with 1.3 mg/l rt-PA in PBS (C, D) for 1 hour. Clot degradation was determined by mass loss (A, C) and by the release of red blood cells–haemoglobin absorbance (B, D). The box plots (square–mean, horizontal line–median, box–interquartile range, whisker–data range) represent data out of three biological replicates each containing three parallels. Significant differences (t-test, p < 0.05) are indicated. Further p values: 0.84 for (C) and 0.16 for (D).

The 2-hours-old clots with mild retraction and 5-hours-old ones with high retraction (S1 Fig) were used for experiments. Because these clots were thought to exhibit inherently different levels of stability, their spontaneous degradation was first determined. Two-hours-old clots showed a more pronounced spontaneous mass loss of the initial weight compared to 5-hours-old clots: 26.9±9.1%, 11.0±5.3%, p<0.001 (Fig 3A, S4 Table). Such trend was confirmed by RBC release: 0.14±0.05, 0.06±0.02, p<0.001 (Fig 3B, S4 Table). The effect of rt-PA on *in vitro* thrombolysis of clots of different age determined by a mass loss against control was: 2-hours-old clots 10.8±7.6%, 5-hours-old clots 11.6±9.9%, p = 0.84 (Fig 3C, S4 Table). Determination of RBC release showed the same results: 0.03±0.02, 0.05±0.04, p = 0.16 (Fig 3D, S4 Table). Thus, the extent of thrombolysis induced by 1.3 mg/l rt-PA was constant regardless of clot age.

### Interaction of rt-PA and heparin

**Fig 4:**
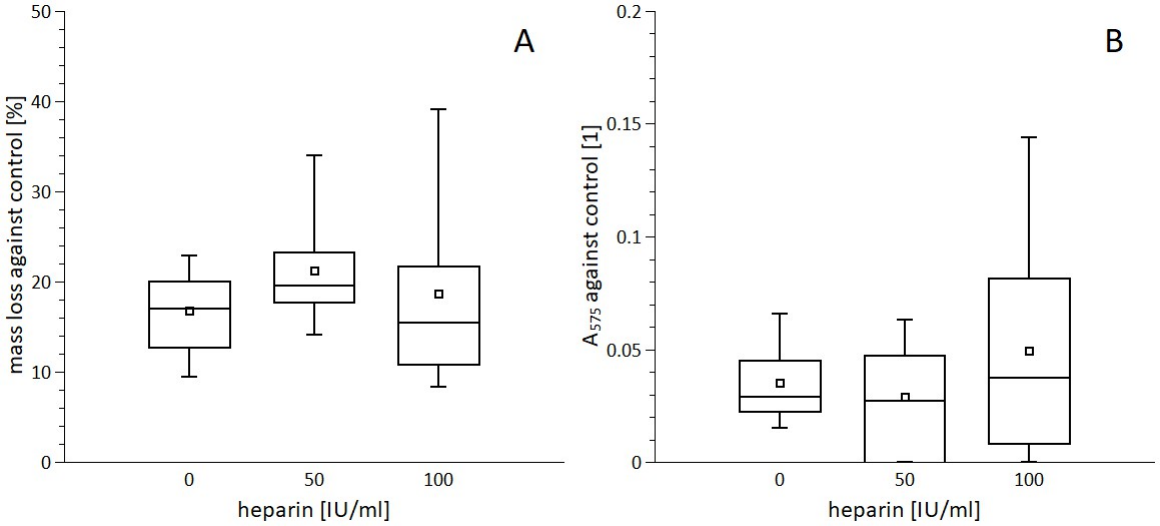


There was no effect of heparin on spontaneous mass loss of clots (S2 File); neither on rt-PA-induced mass loss, documented by the same clot mass loss against control: rt-PA + 0 IU/ml heparin 16.8±4.5%, rt-PA + 50 IU/ml heparin 21.2±6.1%, rt-PA + 100 IU/ml heparin 18.6±9.8% (0 vs 50 IU/mL p = 0.20, 0 vs 100 IU/mL p = 0.60, 50 vs 100 IU/mL p = 0.45; Fig 4A, S5 Table). Determination of RBC release provided the same results: 0.04±0.02, 0.03±0.03, 0.05±0.05 (0 vs 50 IU/mL p = 0.71, 0 vs 100 IU/mL p = 0.39, 50 vs 100 IU/mL p = 0.23; Fig 4B, S5 Table).

## Discussion

The primary and noteworthy outcome of our study is the observation that thrombolysis occurs more rapidly in smaller clots compared to larger ones, becoming progressively less efficient for clot sizes of 50 μl and beyond. Specifically, 30 μl clots exhibited approximately 25% faster relative mass loss compared to 50 μl clots. The probability of achieving a similar extent of thrombolysis gradually decreased with increasing clot size, primarily due to consistent rt-PA-induced red blood cell (RBC) release observed for clot sizes of 50 μl and larger. Consequently, a clot size of 50 μl seems to serve as a threshold, beyond which rt-PA efficiency diminishes. It’s worth noting that previous in vitro studies on rt-PA thrombolysis indicated a linear dependence on clot size without a discernible threshold [23, 24]. However, these studies simulated non-occlusive clots under flow conditions, whereas our data pertains to complete occlusions (see ’Relevance of the model and its limitations’ section).

The identified threshold clot size of 50 μl corresponds to approximately a 7 mm long occlusion of the M1 segment of the middle cerebral artery, a common site of occlusion given the artery diameter. Our results align well with clinical findings: two major clinical studies documented that occluded artery sections longer than 8 mm were unlikely to be cleared with intravenous rt-PA treatment [7, 8]. The finding of a threshold for clot degradation after which the rt-PA becomes increasingly less efficient indicates the presence of a mechanism that restricts thrombolysis. Understanding such a mechanism could lead to improvement in clinical thrombolysis.

Hypothetically, the thrombolysis rate could be dependent on clot surface area. Accordingly, a gradual increase of the RBC release should have been observed with increasing clot size, as the dependence of the surface area of an approximately spherical clot on its size has no upper limit. The contradictory observation in this work (i.e., same RBC release after reaching clot size of 50 μl) may be correlated with limited diffusion of rt-PA in the clot due to its tight binding to fibrin [25]. Such evidence highlights the need for future thrombolytics with improved clot-penetrating capabilities.

Notably, our data reveals that altering rt-PA concentration by a factor of 10 results in only a twofold change in clot degradation. This indicated that the therapeutic level of rt-PA at 1.3 mg/l was rather close to saturation to induce thrombolysis. Our observation in principle agrees with previous work using pure fibrin gels, where t-PA caused a feedback inhibition on plasmin-mediated fibrinolysis [26]. Furthermore, a work dealing with clots prepared *in vitro* from blood out of cardiac patients indicated a very similar course of thrombolysis in dependence on rt-PA concentration [27]. This agreement extends to clinical data as well and our results can provide a partial explanation of clinical observations highlighting the importance of our research. First, it was shown that variation of the dosage within ±20% of the standard 0.9 mg/kg did not affect the clinical outcome [11]. The ENCHANTED clinical trial proved that the 33% reduction of rt-PA dosage did not change the clinical outcome as well [12]. According to our model, the 20% or 33% decrease of rt-PA would result in about 10% or 12% decrease of rt-PA-induced thrombolysis, respectively, indicating further factors behind the clinical observation. The explanation of our finding could be attributed to two potential mechanisms. Excess rt-PA may remove the clot-bound plasminogen necessary for thrombolysis [28]; or the direct negative effect of rt-PA on plasmin action at high concentrations [26].

Our study introduces novel implications regarding the role of clot age in limiting thrombolysis. Although we observed faster spontaneous thrombolysis in 2-hours-old clots than in 5-hours-old clots, the net rt-PA-induced thrombolysis was independent of clot age. Consequently, total thrombolysis (spontaneous and rt-PA-induced components) was higher for younger clots. In principle, the presented data are in agreement with an *in vitro* study with clots prepared from human blood by Mercado-Shekhar et al. The mildly retracted clots (equivalent to 2-hours-old clots in our study) showed higher total thrombolysis than highly retracted clots (equivalent to 5-hours-old clots in our study). The net rt-PA-induced thrombolysis was comparable among these clots [22]. However, there was a disagreement with an *in vitro* study with clots prepared from horse blood where retraction was allowed. The extent of net rt-PA-induced thrombolysis was lower for younger clots though the clot structure was very similar to our case [29]. This could be attributed to species differences. In our study, the more rapid total degradation in younger clots suggests that early administration of rt-PA could be beneficial. Indeed, our finding is in agreement with the results of three clinical studies focusing on artery recanalization during stroke treatment. They concluded that early vessel reopening was dependent on early rt-PA administration [13–15]. The EXTEND clinical study demonstrated that rt-PA could be efficient up to 9 hours after the onset of symptoms in patients who had salvageable regions of the brain [10]. Our results support these findings, emphasizing that the degradability of older clots is attributed to the specific contribution of rt-PA which appears to be independent of clot age. The explanation of higher susceptibility to thrombolysis in younger clots could be attributed to their lower extent of retraction and resulting in lower stiffness [22]. The independence of rt-PA action on clot age could be attributed to the tight binding of rt-PA to fibrin [25] as this is likely a more restrictive factor of the rt-PA entrance to the clot rather than reduction of porosity [22] with clot ageing.

Combining rt-PA with heparin could be a valuable clinical concept [16, 17] but our data do not confirm the positive effect of heparin on rt-PA enzymatic action if applied at clinically relevant concentration. Such observation is contradictory to some early studies, which indicated that heparin could activate rt-PA at concentrations up to 50 IU/ml. In these studies, however, a homogenous reaction mixture was used, which is far more simplistic than an inhomogeneous and complex clot structure [30–32]. On the other hand, heparin may increase rt-PA-induced thrombolysis if present in clots since their formation. The mechanism behind this has been rather attributed to the lower crosslinking of fibrin in clots [33]. Therefore, the novel finding of our work is that the positive effect of heparin on the enzymatic action of rt-PA can be clearly excluded at clinically relevant concentration of rt-PA. In contrast, this was not the case for urokinase since this enzyme is remarkably activated with heparin [30, 34].

### Relevance of the model and limitations

The in vitro model employed RBC-dominant clots, a common cause of brain vasculature occlusions [35]. The size of clots 30 to 150 μl included in the presented study was relevant to ischemic stroke [7, 8]. Their structure was rather compacted but individual elements like RBCs and fibrin fibres were still discernible (See S3 Fig). Such observations indicated reasonable maturity of clots and their relevance to ischemic stroke [36]. The degree of clot compaction was even higher than in a comparable *in vitro* study, though fewer fibrin filaments were observed [21]. The compacted clot structure was well correlated with a high level of retraction permitted by clot preparation in borosilicate glass tubes, again comparable to relevant *in vitro* literature [21, 22]. The concentration of rt-PA was chosen so that it matched the therapeutic level in an average patient. Thrombolysis was evaluated after one hour which corresponded to the one-hour long infusion of rt-PA in a clinical setting [19]. As a readout, clot mass decrease [37] and RBC release corrected to turbidity was used. The described model is related to complete occlusion with a lack of flow. Therefore, clot degradation was mediated by thrombolysis only, without any contribution of the erosive effect of flow [23, 24]. The extent of rt-PA-induced clot lysis (10 to 30% mass loss, see Figs 1–4) was comparable to the one *in vivo* (about 1/3 per hour [38]). Moreover, with the static model, it was found that the no-response-to-rt-PA rate was about 30%, which compared well to 25% reported in the literature [6]. Compared to competing approaches, our model balanced pathophysiological relevance to complete vessel occlusions, data reliability and simplicity [6, 23, 24, 39].

However, our study has several limitations. The data should not be extrapolated to incomplete occlusions as residual flow aids mechanical clot degradation [23, 24]. The absence of plasminogen in the incubation medium in our model is generally no problem for rt-PA-mediated thrombolysis because of a sufficient quantity of plasminogen in the clot. However, it may hamper the thrombolysis mediated by agents with high demand for plasminogen–e.g. staphylokinase [40]. Additionally, our deliberately chosen incubation medium lacked key thrombolysis inhibitors, including plasminogen activator inhibitor 1 and antiplasmin [25]. Notably, the levels of these compounds naturally vary among blood donors. If experiments were conducted in a more naturalistic incubation medium, such as plasma or heparinized blood, we anticipate a greater variability in the obtained data. Another potential limitation is the use of blood from healthy donors. In clinical practice, stroke patients suffer from comorbidities (hypertension, diabetes mellitus, dyslipidemia, other cardiovascular diseases, etc.) [14]. With the exception of diabetes mellitus, these comorbidities do not directly affect the function of rt-PA [41]. Hence, we do not expect major differences in results that would be obtained using blood from patients without diabetes mellitus.

In conclusion, our work has contributed valuable insights into factors limiting rt-PA-induced thrombolysis. Significantly, we identified clot size as a paramount determinant, unveiling a critical threshold beyond which rt-PA efficacy diminishes—clots larger than 50 μl proved challenging to degrade. This highlights a compelling need for further research to enhance thrombolytic effectiveness in the context of large clots. Additionally, our findings underscored that increased rt-PA concentration or the addition of heparin yielded limited or no improvement in thrombolytic efficacy, respectively. Notably, younger clots exhibited higher susceptibility to thrombolysis, lending support to the consideration of reducing the time from stroke onset to rt-PA treatment in clinical practice.

## Supporting information

S1 Fig Length of whole blood clots from different amounts of blood in the in vitro middle cerebral artery model (diameter 3.1 mm). In order to determine the clot size as well as the hypothetical length of an occlusion in the middle cerebral artery (average internal diameter 3.1 mm), the clots were gently transferred into a tubing with corresponding dimensions. The length of an occluded section of the middle cerebral artery was determined as the average distance between clot ends (N = 3, see figure). The volume was calculated using the formula for a cylinder using the previously estimated length. (a) Amount of blood 100 μl–length 4 mm–clot size 30 μl, (b) Amount of blood 200 μl–length 7 mm–clot size 50 μl, (c) Amount of blood 300 μl–length 12 mm–clot size 90 μl, (d) Amount of blood 400 μl–length 20 mm–clot size 150 μl.
(TIF)

S2 Fig Images of the tubes during clot preparation. Clots were prepared in borosilicate glass (Pyrex) which was already shown to allow consistent clot retraction (Sutton et al. 2013; Mercado-Shekhar et al. 2018). The extent of retraction in our work was directed by the time of clotting instead of the use of different types of glass (e.g. soda lime glass). Immediately after filling with 200 μl blood without anticoagulants, 2 and 5 hours since the start of clotting at room temperature.
(TIF)

S3 Fig Five-hours-old clots were subjected to scanning electron microscopy. To visualize the microscopic structure of the clots, the scanning electron microscopy technique was used. Clots were fixed with 3% glutaraldehyde in 0.1M cacodylate buffer. The clots were washed three times with 0.1M cacodylate buffer, dehydrated using ascending ethanol grade and dried in a critical point dryer (CPD 030, BAL-TEC Inc., Liechtenstein) using liquid carbon dioxide. Dried samples were sputtered with gold in sputter coater (SCD 040, Balzers Union Limited, Liechtenstein) and observed in a scanning electron microscope (VEGA TS 5136 XM, Tescan Group, a.s., Czech Republic) using a secondary emission detector and 20 kV acceleration voltage. There were three major structures found in the lab-made clots. The outer envelope of the clot formed in contact with the borosilicate glass vessel during the clotting process was composed of the thin and dense layer of fibrin filaments. The upper part exposed to the residual serum was formed by thick loose fibrin filaments with other particles adhered to them and mostly erythrocytes with the normal shape. The vast inside part of the clot was formed by tightly packed polyhedral-shaped erythrocytes and thick fibrin filaments. Images show major structures of the clot. Typical images out of three biological replicates are presented.
(TIF)

S1 Table Stability of clots in different environments (PBS, 0.9% NaCl, heparinized blood). Clot lysis is expressed as relative clot mass loss.
(PDF)

S2 Table Thrombolysis of clots of different sizes (30, 50, 90 and 150 μl). Clot lysis is expressed as relative clot mass loss against control and RBC release against control.
(PDF)

S3 Table Thrombolysis induced by rt-PA concentration of 0.13, 1.3 and 13 mg/l. Clot lysis is expressed as relative clot mass loss against control and RBC release against control.
(PDF)

S4 Table Thrombolysis of clots of different ages (2- and 5-hours-old). Clot lysis is expressed as relative clot mass loss against control and RBC release against control.
(PDF)

S5 Table Thrombolysis induced by rt-PA (1.3 mg/l) and heparin (0, 50, and 100 IU/ml). Clot lysis is expressed as relative clot mass loss against control and RBC release against control.
(PDF)

S1 File Clot type and incubation medium. (PDF)

S2 File Complete data. (XLSX)

## References

[1] Lopez AD, Mathers CD, Ezzati M, Jamison DT, Murray CJL (2006) Global and regional burden of disease and risk factors, 2001: systematic analysis of population health data. Lancet 367: 1747–1757. doi: 10.1016/S0140-6736(06)68770-9 16731270

[2] Lloyd-Jones D, Adams R, Carnethon M, De Simone G, Ferguson TB, et al. (2009) 22. Glossary. Circulation 119: e21–e181. 19075105 10.1161/CIRCULATIONAHA.108.191261

[3] Powers WJ, Derdeyn CP, Biller J, Coffey CS, Hoh BL, et al. (2015) 2015 AHA/ASA Focused Update of the 2013 Guidelines for the Early Management of Patients With Acute Ischemic Stroke Regarding Endovascular Treatment, A Guideline for Healthcare Professionals From the American Heart Association/American Stroke Association. Stroke 46: 3020–3035. 26123479 10.1161/STR.0000000000000074

[4] Hacke W, Kaste M, Bluhmki E, Brozman M, Dávalos A, et al. (2008) Thrombolysis with alteplase 3 to 4.5 hours after acute ischemic stroke. N Engl J Med 359: 1317–1329. doi: 10.1056/NEJMoa0804656 18815396

[5] NINDS_Group (1995) Tissue plasminogen activator for acute ischemic stroke. N Engl J Med 333: 1581–1588. doi: 10.1056/NEJM199512143332401 7477192

[6] Meunier JM, Wenker E, Lindsell CJ, Shaw GJ (2013) Individual lytic efficacy of recombinant tissue plasminogen activator in an in vitro human clot model: rate of “nonresponse”. Acad Emerg Med 20: 449–455. doi: 10.1111/acem.12133 23672358 PMC3658149

[7] Kamalian S, Morais LT, Pomerantz SR, Aceves M, Sit SP, et al. (2013) Clot length distribution and predictors in anterior circulation stroke: implications for intra-arterial therapy. Stroke 44: 3553–3556. doi: 10.1161/STROKEAHA.113.003079 24105699 PMC3927722

[8] Riedel CH, Zimmermann P, Jensen-Kondering U, Stingele R, Deuschl G, et al. (2011) The importance of size: successful recanalization by intravenous thrombolysis in acute anterior stroke depends on thrombus length. Stroke 42: 1775–1777. doi: 10.1161/STROKEAHA.110.609693 21474810

[9] Alexandrov AV, Demchuk AM, Felberg RA, Christou I, Barber PA, et al. (2000) High rate of complete recanalization and dramatic clinical recovery during tPA infusion when continuously monitored with 2-MHz transcranial Doppler monitoring. Stroke 31: 610–614. doi: 10.1161/01.str.31.3.610 10700493

[10] Ma H, Campbell BC, Parsons MW, Churilov L, Levi CR, et al. (2019) Thrombolysis Guided by Perfusion Imaging up to 9 Hours after Onset of Stroke. N Engl J Med 380: 1795–1803. doi: 10.1056/NEJMoa1813046 31067369

[11] Aulicky P, Rabinstein A, Seet RC, Neumann J, Mikulik R (2013) Dosing of tissue plasminogen activator often differs from 0.9 mg/kg, but does not affect the outcome. J Stroke Cerebrovasc Dis 22: 1293–1297. doi: 10.1016/j.jstrokecerebrovasdis.2012.10.010 23246191

[12] Anderson CS, Robinson T, Lindley RI, Arima H, Lavados PM, et al. (2016) Low-dose versus standard-dose intravenous alteplase in acute ischemic stroke. N Engl J Med 374: 2313–2323. doi: 10.1056/NEJMoa1515510 27161018

[13] Kimura K, Iguchi Y, Shibazaki K, Aoki J, Watanabe M, et al. (2010) Early stroke treatment with IV t-PA associated with early recanalization. J Neurol Sci 295: 53–57. doi: 10.1016/j.jns.2010.05.012 20570289

[14] Tsivgoulis G, Saqqur M, Sharma VK, Brunser A, Eggers J, et al. (2020) Timing of Recanalization and Functional Recovery in Acute Ischemic Stroke. J Stroke 22: 130. doi: 10.5853/jos.2019.01648 32027798 PMC7005347

[15] Emberson J, Lees KR, Lyden P, Blackwell L, Albers G, et al. (2014) Effect of treatment delay, age, and stroke severity on the effects of intravenous thrombolysis with alteplase for acute ischaemic stroke: a meta-analysis of individual patient data from randomised trials. Lancet 384: 1929–1935. doi: 10.1016/S0140-6736(14)60584-5 25106063 PMC4441266

[16] Ruff IM, Jindal JA (2015) Use of Heparin in Acute Ischemic Stroke: Is There Still a Role? Curr Atheroscleros Rep 17: 51. doi: 10.1007/s11883-015-0528-3 26194057

[17] Mikulik R, Wahlgren N (2015) Treatment of acute stroke: an update. J Intern Med 278: 145–165. doi: 10.1111/joim.12387 26130489

[18] Thalerová S, Pešková M, Kittová P, Gulati S, Víteček J, et al. (2021) Effect of Apixaban Pretreatment on Alteplase-Induced Thrombolysis: An In Vitro Study. Front Pharmacol: 2449. doi: 10.3389/fphar.2021.740930 34603054 PMC8479181

[19] Acheampong P, Ford GA (2012) Pharmacokinetics of alteplase in the treatment of ischaemic stroke. Expert Opin Drug Metab Toxicol 8: 271–281. doi: 10.1517/17425255.2012.652615 22248305

[20] Hirsh J, Anand SS, Halperin JL, Fuster V (2001) Guide to anticoagulant therapy: Heparin: a statement for healthcare professionals from the American Heart Association. Circulation 103: 2994–3018. doi: 10.1161/01.cir.103.24.2994 11413093

[21] Sutton JT, Ivancevich NM, Perrin SR, Vela DC, Holland CK (2013) Clot retraction affects the extent of ultrasound-enhanced thrombolysis in an ex vivo porcine thrombosis model. Ultrasound Med Biol 39: 813–824. doi: 10.1016/j.ultrasmedbio.2012.12.008 23453629 PMC3618502

[22] Mercado-Shekhar KP, Kleven RT, Rivera HA, Lewis R, Karani KB, et al. (2018) Effect of clot stiffness on recombinant tissue plasminogen activator lytic susceptibility in vitro. Ultrasound Med Biol 44: 2710–2727. doi: 10.1016/j.ultrasmedbio.2018.08.005 30268531 PMC6551517

[23] Bajd F, Serša I (2012) A concept of thrombolysis as a corrosion-erosion process verified by optical microscopy. Microcirculation 19: 632–641. doi: 10.1111/j.1549-8719.2012.00198.x 22612378

[24] Bajd F, Vidmar J, Blinc A, Sersa I (2010) Microscopic clot fragment evidence of biochemo-mechanical degradation effects in thrombolysis. Thromb Res 126: 137–143. doi: 10.1016/j.thromres.2010.04.012 20580981

[25] Diamond SL (1999) Engineering design of optimal strategies for blood clot dissolution. Annu Rev Biomed Eng 1: 427–462. doi: 10.1146/annurev.bioeng.1.1.427 11701496

[26] Wu JH, Diamond SL (1995) Tissue plasminogen activator (tPA) inhibits plasmin degradation of fibrin. A mechanism that slows tPA-mediated fibrinolysis but does not require alpha 2-antiplasmin or leakage of intrinsic plasminogen. J Clin Invest 95: 2483–2490. doi: 10.1172/JCI117949 7769094 PMC295930

[27] Musselman DR, Tate DA, Oberhardt BJ, Abruzzini AF, Blauwet MB, et al. (1994) Differences in clot lysis among patients demonstrated in vitro with three thrombolytic agents (tissue-type plasminogen activator, streptokinase and urokinase). The American journal of cardiology 73: 544–549. doi: 10.1016/0002-9149(94)90330-1 8147298

[28] Torr SR, Nachowiak DA, Fujii S, Sobel BE (1992) “Plasminogen steal” and clot lysis. J Am Coll Cardiol 19: 1085–1090. doi: 10.1016/0735-1097(92)90300-c 1532403

[29] Zhou Y, Murugappan SK, Sharma VK (2014) Effect of clot aging and cholesterol content on ultrasound-assisted thrombolysis. Translational stroke research 5: 627–634. doi: 10.1007/s12975-014-0332-3 24488442

[30] Dosne A, Bendetowicz A, Kher A, Samama M (1988) Marked potentiation of the plasminogenolytic activitiy of pro-urokinase by unfractionated heparin and a low molecular-weight heparin. Thromb Res 51: 627–630. 2847356 10.1016/0049-3848(88)90146-6

[31] Andrade-Gordon P, Strickland S (1986) Interaction of heparin with plasminogen activators and plasminogen: effects on the activation of plasminogen. Biochemistry 25: 4033–4040. doi: 10.1021/bi00362a007 2943315

[32] Paques E-P, Stӧhr H-A, Heimburger N (1986) Study on the mechanism of action of heparin and related substances on the fibrinolytic system: Relationsship between plasminogen activators and heparin. Thromb Res 42: 797–807. doi: 10.1016/0049-3848(86)90116-7 3088755

[33] Tamao Y, Yamamoto T, Kikumoto R, Hara H, Itoh J, et al. (1986) Effect of a selective thrombin inhibitor MCI-9038 on fibrinolysis in vitro and in vivo. Thrombosis and haemostasis 56: 028–034. 2877508

[34] Del Zoppo GJ, Higashida RT, Furlan AJ, Pessin MS, Rowley HA, et al. (1998) PROACT: a phase II randomized trial of recombinant pro-urokinase by direct arterial delivery in acute middle cerebral artery stroke. Stroke 29: 4–11. 9445320 10.1161/01.str.29.1.4

[35] Liebeskind DS, Sanossian N, Yong WH, Starkman S, Tsang MP, et al. (2011) CT and MRI early vessel signs reflect clot composition in acute stroke. Stroke 42: 1237–1243. doi: 10.1161/STROKEAHA.110.605576 21393591 PMC3094751

[36] Mehta BP, Nogueira RG (2012) Should clot composition affect choice of endovascular therapy? Neurology 79: S63–7. doi: 10.1212/WNL.0b013e3182695859 23008415

[37] Prasad S, Kashyap RS, Deopujari JY, Purohit HJ, Taori GM, et al. (2006) Development of an in vitro model to study clot lysis activity of thrombolytic drugs. Thromb J 4: 1–4. 16968529 10.1186/1477-9560-4-14 PMC1570448

[38] Kim YD, Nam HS, Kim SH, Kim EY, Song D, et al. (2015) Time-dependent thrombus resolution after tissue-type plasminogen activator in patients with stroke and mice. Stroke 46: 1877–1882. doi: 10.1161/STROKEAHA.114.008247 25967573

[39] Datta S, Coussios C-C, Ammi AY, Mast TD, de Courten-Myers GM, et al. (2008) Ultrasound-enhanced thrombolysis using Definity as a cavitation nucleation agent. Ultrasound Med Biol 34: 1421–1433. doi: 10.1016/j.ultrasmedbio.2008.01.016 18378380 PMC2945910

[40] Nikitin D, Choi S, Mican J, Toul M, Ryu W-S, et al. (2021) Development and Testing of Thrombolytics in Stroke. J Stroke 23: 12. doi: 10.5853/jos.2020.03349 33600700 PMC7900387

[41] Zangerle A, Kiechl S, Spiegel M, Furtner M, Knoflach M, et al. (2007) Recanalization after thrombolysis in stroke patients: predictors and prognostic implications. Neurology 68: 39–44. doi: 10.1212/01.wnl.0000250341.38014.d2 17200490

