# Supplementary figures and images for: Factors influencing the efficacy of recombinant tissue plasminogen activator: Implications for ischemic stroke treatment
Source: PLoS One. 2024 Jun 6;19(6):e0302269. doi: 10.1371/journal.pone.0302269 (PMC11156348; doi:10.1371/journal.pone.0302269)

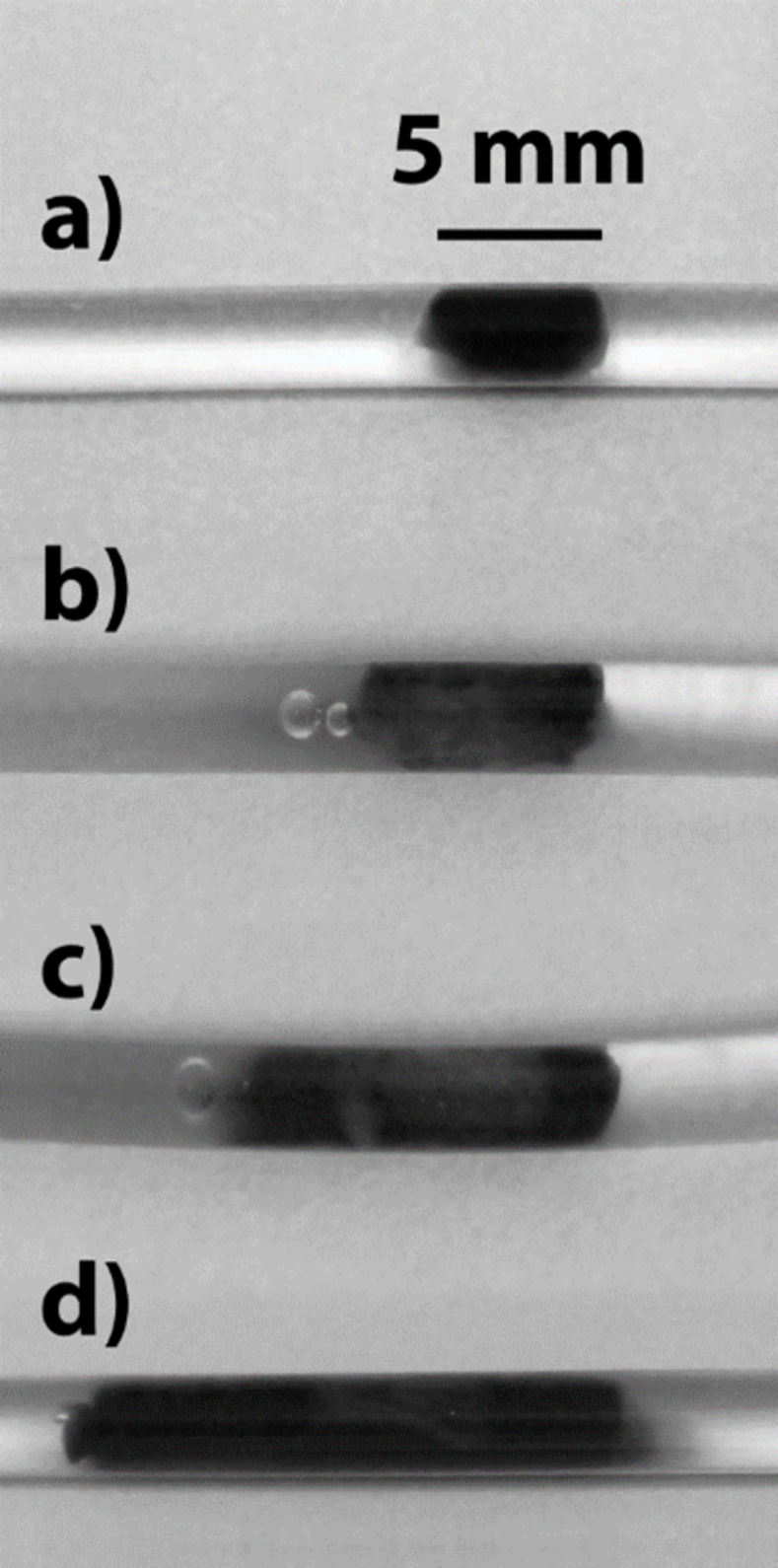

Supplement: S1 Fig — In order to determine the clot size as well as the hypothetical length of an occlusion in the middle cerebral artery (average internal diameter 3.1 mm), the clots were gently transferred into a tubing with corresponding dimensions. The length of an occluded section of the middle cerebral artery was determined as the average distance between clot ends (N = 3, see figure). The volume was calculated using the formula for a cylinder using the previously estimated length. (a) Amount of blood 100 μl–length 4 mm–clot size 30 μl, (b) Amount of blood 200 μl–length 7 mm–clot size 50 μl, (c) Amount of blood 300 μl–length 12 mm–clot size 90 μl, (d) Amount of blood 400 μl–length 20 mm–clot size 150 μl. (TIF) [file pone.0302269.s001.tif]

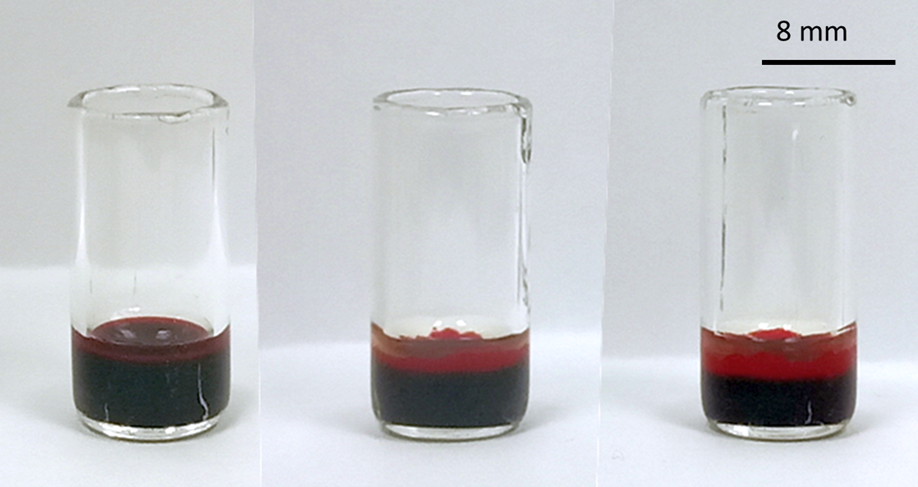

Supplement: S2 Fig — Clots were prepared in borosilicate glass (Pyrex) which was already shown to allow consistent clot retraction (Sutton et al. 2013; Mercado-Shekhar et al. 2018). The extent of retraction in our work was directed by the time of clotting instead of the use of different types of glass (e.g. soda lime glass). Immediately after filling with 200 μl blood without anticoagulants, 2 and 5 hours since the start of clotting at room temperature. (TIF) [file pone.0302269.s002.tif]

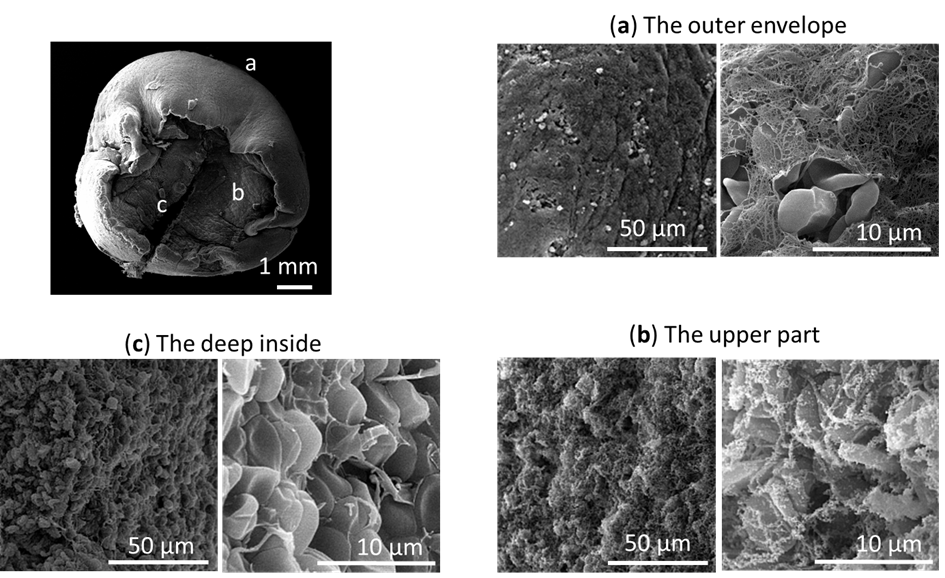

Supplement: S3 Fig — To visualize the microscopic structure of the clots, the scanning electron microscopy technique was used. Clots were fixed with 3% glutaraldehyde in 0.1M cacodylate buffer. The clots were washed three times with 0.1M cacodylate buffer, dehydrated using ascending ethanol grade and dried in a critical point dryer (CPD 030, BAL-TEC Inc., Liechtenstein) using liquid carbon dioxide. Dried samples were sputtered with gold in sputter coater (SCD 040, Balzers Union Limited, Liechtenstein) and observed in a scanning electron microscope (VEGA TS 5136 XM, Tescan Group, a.s., Czech Republic) using a secondary emission detector and 20 kV acceleration voltage. There were three major structures found in the lab-made clots. The outer envelope of the clot formed in contact with the borosilicate glass vessel during the clotting process was composed of the thin and dense layer of fibrin filaments. The upper part exposed to the residual serum was formed by thick loose fibrin filaments with other particles adhered to them and mostly erythrocytes with the normal shape. The vast inside part of the clot was formed by tightly packed polyhedral-shaped erythrocytes and thick fibrin filaments. Images show major structures of the clot. Typical images out of three biological replicates are presented. (TIF) [file pone.0302269.s003.tif]
