# Supplementary material for: Factors influencing the efficacy of recombinant tissue plasminogen activator: Implications for ischemic stroke treatment
Source: PLoS One. 2024 Jun 6;19(6):e0302269. doi: 10.1371/journal.pone.0302269 (PMC11156348; doi:10.1371/journal.pone.0302269)
Supplement: S1 Table — Clot lysis is expressed as relative clot mass loss. (PDF) [file pone.0302269.s004.pdf]

**Clot mass loss**	Mean [%]	Median [%]	SD [%]	Lower CI (95%) [%]	Upper CI (95%) [%]	Minimum [%]	Maximum [%]	Count
PBS	18.2	17.1	7.0	13.8	22.7	7.8	30.8	12
0.9% NaCl	15.2	16.0	4.5	12.3	18.0	7.1	20.2	12
heparinized blood	17.0	18.0	6.4	12.9	21.0	3.6	24.5	12

SD, standard deviation; CI, confidence interval
