# Supplementary material for: Factors influencing the efficacy of recombinant tissue plasminogen activator: Implications for ischemic stroke treatment
Source: PLoS One. 2024 Jun 6;19(6):e0302269. doi: 10.1371/journal.pone.0302269 (PMC11156348; doi:10.1371/journal.pone.0302269)
Supplement: S2 Table — Clot lysis is expressed as relative clot mass loss against control and RBC release against control. (PDF) [file pone.0302269.s005.pdf]

**Clot mass loss against control**	Mean [%]	Median [%]	SD [%]	Lower CI (95%) [%]	Upper CI (95%) [%]	Minimum [%]	Maximum [%]	Count
30 µl	20.4	11.5	16.1	8.0	32.8	7.8	55.9	9
50 µl	16.2	15.0	9.5	11.4	20.9	0.0	31.7	18
90 µl	13.2	12.5	6.0	10.2	16.2	2.4	24.1	18
150 µl	6.5	5.3	2.7	4.5	8.6	3.9	12.6	9
**RBC release against control**	Mean [1]	Median [1]	SD [1]	Lower CI (95%) [1]	Upper CI (95%) [1]	Minimum [1]	Maximum [1]	Count
30 µl	0.03	0.03	0.01	0.02	0.04	0.01	0.06	9
50 µl	0.07	0.07	0.03	0.05	0.08	0.01	0.12	16
90 µl	0.08	0.08	0.05	0.05	0.10	0.01	0.17	14
150 µl	0.07	0.08	0.03	0.05	0.08	0.03	0.09	9

SD, standard deviation; CI, confidence interval
