# Supplementary material for: Factors influencing the efficacy of recombinant tissue plasminogen activator: Implications for ischemic stroke treatment
Source: PLoS One. 2024 Jun 6;19(6):e0302269. doi: 10.1371/journal.pone.0302269 (PMC11156348; doi:10.1371/journal.pone.0302269)
Supplement: S3 Table — Clot lysis is expressed as relative clot mass loss against control and RBC release against control. (PDF) [file pone.0302269.s006.pdf]

**Clot mass loss against control**	Mean [%]	Median [%]	SD [%]	Lower CI (95%) [%]	Upper CI (95%) [%]	Minimum [%]	Maximum [%]	Count
0.13 mg/l	12.5	12.4	4.1	9.3	15.6	7.7	18.8	9
1.3 mg/l	24.0	22.3	4.8	20.3	27.6	16.4	32.0	9
13 mg/l	30.3	30.1	4.0	27.2	33.4	23.2	37.5	9
**RBC release against control**	Mean [1]	Median [1]	SD [1]	Lower CI (95%) [1]	Upper CI (95%) [1]	Minimum [1]	Maximum [1]	Count
0.13 mg/l	0.04	0.04	0.02	0.03	0.06	0.01	0.08	9
1.3 mg/l	0.07	0.06	0.03	0.04	0.09	0.01	0.12	9
13 mg/l	0.11	0.12	0.03	0.08	0.14	0.07	0.13	5

SD, standard deviation; CI, confidence interval
