# Supplementary material for: Factors influencing the efficacy of recombinant tissue plasminogen activator: Implications for ischemic stroke treatment
Source: PLoS One. 2024 Jun 6;19(6):e0302269. doi: 10.1371/journal.pone.0302269 (PMC11156348; doi:10.1371/journal.pone.0302269)
Supplement: S4 Table — Clot lysis is expressed as relative clot mass loss against control and RBC release against control. (PDF) [file pone.0302269.s007.pdf]

**Clot mass loss**	Mean [%]	Median [%]	SD [%]	Lower CI (95%) [%]	Upper CI (95%) [%]	Minimum [%]	Maximum [%]	Count
2 hours	26.9	21.7	9.1	20.0	33.9	19.7	43.1	9
5 hours	11.0	10.6	5.3	6.9	15.1	3.4	17.5	9
**RBC release**	Mean [1]	Median [1]	SD [1]	Lower CI (95%) [1]	Upper CI (95%) [1]	Minimum [1]	Maximum [1]	Count
2 hours	0.14	0.14	0.05	0.11	0.18	0.09	0.20	9
5 hours	0.06	0.07	0.02	0.05	0.08	0.04	0.09	9

SD, standard deviation; CI, confidence interval

**Clot mass loss against control**	Mean [%]	Median [%]	SD [%]	Lower CI (95%) [%]	Upper CI (95%) [%]	Minimum [%]	Maximum [%]	Count
2 hours	10.8	8.2	7.6	4.9	16.6	0.0	20.6	9
5 hours	11.6	11.0	9.9	4.0	19.2	0.0	31.7	9
**RBC release against control**	Mean [1]	Median [1]	SD [1]	Lower CI (95%) [1]	Upper CI (95%) [1]	Minimum [1]	Maximum [1]	Count
2 hours	0.03	0.03	0.02	0.02	0.05	0.01	0.06	9
5 hours	0.05	0.06	0.04	0.02	0.08	0.00	0.11	9

SD, standard deviation; CI, confidence interval
