# Supplementary material for: Factors influencing the efficacy of recombinant tissue plasminogen activator: Implications for ischemic stroke treatment
Source: PLoS One. 2024 Jun 6;19(6):e0302269. doi: 10.1371/journal.pone.0302269 (PMC11156348; doi:10.1371/journal.pone.0302269)
Supplement: S5 Table — Clot lysis is expressed as relative clot mass loss against control and RBC release against control. (PDF) [file pone.0302269.s008.pdf]

**Clot mass loss against control**	Mean [%]	Median [%]	SD [%]	Lower CI (95%) [%]	Upper CI (95%) [%]	Minimum [%]	Maximum [%]	Count
0 IU/ml	16.8	17.0	4.5	13.4	20.3	9.4	22.9	9
50 IU/ml	21.2	19.6	6.1	16.5	25.9	14.1	34.0	9
100 IU/ml	18.6	15.4	9.8	11.1	26.1	8.3	39.1	9
**RBC release against control**	Mean [1]	Median [1]	SD [1]	Lower CI (95%) [1]	Upper CI (95%) [1]	Minimum [1]	Maximum [1]	Count
0 IU/ml	0.04	0.03	0.02	0.02	0.05	0.02	0.07	9
50 IU/ml	0.03	0.03	0.03	0.01	0.05	0.00	0.06	9
100 IU/ml	0.05	0.04	0.05	0.01	0.09	0.00	0.14	9

SD, standard deviation; CI, confidence interval
