# Supplementary material for: Factors influencing the efficacy of recombinant tissue plasminogen activator: Implications for ischemic stroke treatment
Source: PLoS One. 2024 Jun 6;19(6):e0302269. doi: 10.1371/journal.pone.0302269 (PMC11156348; doi:10.1371/journal.pone.0302269)
Supplement: S1 File — (PDF) [file pone.0302269.s009.pdf]

Clot type and incubation medium

The thrombolysis model used in this study was based on red blood cell dominant clots which are frequently present *in vivo* Liebeskind et al. (2011) and can be prepared in a repeatable manner. First of all, clot stability (i.e. spontaneous thrombolysis) was addressed; which was found to be essentially the same in all test environments (0.9% NaCl, PBS buffer, plasma heparinized blood). Since simple media such as 0.9% NaCl and PBS induced the same degree of spontaneous thrombolysis visible in plasma and heparinized blood, they provide an obvious advantage for the maintenance of simple experimental conditions and future work in the field. Further, the absence of thrombolysis inhibitors such as plasminogen activator inhibitor 1 and antiplasmin, which can cause another level of variability, aided a rigorous focus on the selected factors (clot size, clot age, rt-PA concentration, heparin level) which were the primary focus of this study.

Reference:

Liebeskind DS, Sanossian N, Yong WH, Starkman S, Tsang MP, Moya AL, et al. CT and MRI early vessel signs reflect clot composition in acute stroke. Stroke. Am Heart Assoc; 2011;42(5):1237–43.
